# The dynamic separation of pallidal neurons into anti-phase oscillatory groups under Parkinsonian conditions in a computational model

**DOI:** 10.1186/1471-2202-15-S1-O18

**Published:** 2014-07-21

**Authors:** Robert Merrison-Hort, Roman Borisyuk

**Affiliations:** 1 School of Computing & Mathematics, Plymouth University, Plymouth, Devon, PL4 8AA, UK

## 

Neurons in the globus pallidus (GP) of urethane anesthetized rats typically display one of four spiking patterns: tonic, non-modulated, firing (the NM group); firing that occurs preferentially during either the active or inactive phases of slow cortical oscillations (TA or TI group, respectively); or silence/quiescence (QU group). In healthy animals the vast majority of neurons are in the non-modulated group. However, under conditions of experimentally-induced Parkinsonism there is a dramatic increase in the number of neurons whose firing patterns show modulation by the slow cortical rhythm, either in-phase or anti-phase [1]. The mechanism that underlies the increased tendency for GP neurons to become entrained by cortical rhythms is unclear, but it may contribute to some of the motor symptoms of Parkinson’s disease.

**Figure 1:**
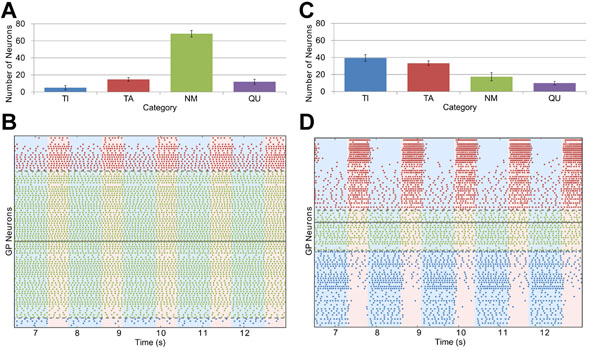
Proportions of neurons in each category (**A**,**C**) and spiking activity sorted by classification confidence (**B**,**D**) for healthy (left) and Parkinsonian (right) parameters.

There are two main pathways from the cortex to the GP: via the inhibitory striatum and via the excitatory subthalamic nucleus (STN), but it is not known how these inputs sculpt the pathological pallidal firing patterns. To study this we developed a neural network model of single compartment conductance-based (Hodgkin-Huxley) pallidal neurons, based on a previous multi-compartment model [2]. The GP neurons received rhythmic input from STN neurons and reciprocal inhibition from each other. Under “healthy” conditions, almost all model GP neurons showed tonic firing that was not significantly modulated by the rhythmic STN input (Figure 1A,B). We attempted to model “Parkinsonian” conditions by increasing the intensity of STN neuron firing and the strength of STN-GP and GP-GP synapses. Under these conditions, two groups of anti-phase oscillatory GP neurons emerged (Figure 1C,D). Our model also includes downregulation of Hyperpolarization activated Cyclic Nucleotide-gated (HCN) channels in response to bursting, since this may contribute to emergence of Parkinsonian activity [3]. We found that this provides better agreement with experimental data but that it is not essential in order for the two groups to appear.

Our results [4] support the hypothesis that oscillatory entrainment occurs primarily via the subthalamic pathway. We find that as a result of the interplay between excitatory input from the STN and mutual inhibition between GP neurons, the network shows a self-organizing dynamical behavior where two groups of neurons (TI and TA) emerge out of a homogeneous population.
